# The polyglutamine-expanded androgen receptor has increased DNA binding and reduced transcriptional activity

**DOI:** 10.1016/j.bbrep.2015.07.014

**Published:** 2015-07-26

**Authors:** Sergey Belikov, Laura C. Bott, Kenneth H. Fischbeck, Örjan Wrange

**Affiliations:** a Department of Cell and Molecular Biology, Karolinska Institutet, SE-17177 Stockholm, Sweden; b Neurogenetics Branch, National Institute of Neurological Disorders and Stroke, National Institutes of Health, Bethesda, MD 20892, USA

**Keywords:** AR, androgen receptor, ARE(s), androgen response element(s), bp, basepair, DMS, dimethylsulphate, MMTV, mouse mammary tumor virus, NTD, N-terminal domain, polyQ, polyglutamine, SBMA, spinal and bulbar muscular atrophy, WB, Western blotting, Androgen receptor, Chromatin, DNA binding, Expanded polyglutamine repeat, Transcription, *Xenopus* oocyte

## Abstract

Expansion of a polyglutamine-encoding trinucleotide CAG repeat in the androgen receptor (AR) to more than 37 repeats is responsible for the X-linked neuromuscular disease spinal and bulbar muscular atrophy (SBMA). Here we evaluated the effect of polyglutamine length on AR function in *Xenopus* oocytes. This allowed us to correlate the nuclear AR concentration to its capacity for specific DNA binding and transcription activation *in vivo*. AR variants with polyglutamine tracts containing either 25 or 64 residues were expressed in *Xenopus* oocytes by cytoplasmic injection of the corresponding mRNAs. The intranuclear AR concentration was monitored in isolated nuclei and related to specific DNA binding as well as transcriptional induction from the hormone response element in the mouse mammary tumor virus (MMTV) promoter. The expanded AR with 64 glutamines had increased capacity for specific DNA binding and a reduced capacity for transcriptional induction as related to its DNA binding activity. The possible mechanism behind these polyglutamine-induced alterations in AR function is discussed.

## Highlights

• Spinal bulbular muscular atrophy is caused by a polyQ expanded androgen receptor.
• Function of AR with expanded polyQ tract was analyzed in *Xenopus* oocytes.
• AR with expanded polyQ tract has increased DNA binding but reduced gene activation.

## 1 Introduction

Androgenic hormones play a vital role in many biological processes in various parts of the body including reproductive organs, kidney, liver, bone, muscle and brain. They exert their role *via* binding to the androgen receptor (AR), a ligand-activated steroid hormone receptor that acts as a transcription factor to control the expression of androgen-dependent genes [1]. The N-terminal transactivation domain (NTD) of the AR protein contains a polymorphic polyglutamine (polyQ) tract which has been linked to spinal and bulbar muscular atrophy (SBMA, Kennedy's disease) [2], a disorder characterized by progressive neuromuscular weakness which develops when its length exceeds 37 residues [3]. The expanded polyQ tract in AR has been demonstrated to alter transcriptional activity of AR in different ways in different cell types. Several studies have shown that AR transcriptional activity inversely correlates with the length of this tract [4], [5], [6], [7], however not all reports are in agreement. Thus, it was shown that AR transcriptional activity is positively affected by increasing polyQ repeat length in skeletal muscle cells [8] thus arguing that the effect of an extended polyQ repeat on AR function is context dependent, for example due to interactions with tissue-specific co-activators. Interestingly, the polyQ repeat length also affects AR stability, possibly because of altered protein folding [7], [9] and recent studies demonstrate beneficial effects on the AR polyQ disease in a mouse model by disrupting the SUMOylation of AR [10].

Although the cause of SBMA is expansion of the CAG repeats in the AR gene the exact disease mechanism remain unclear. We decided to use *Xenopus* oocytes to look more closely at the function of the AR with an expanded polyQ tract. The large size of these cells allows quantification of intranuclear receptor concentration, sequence specific DNA binding and AR target gene activation [11]. As a gene target we used the enhancer and promoter of the mouse mammary tumor virus (MMTV) since this is a useful model system for studies of hormone regulation by glucocorticoids [12] progestins and androgens [11].

An advantage of the *Xenopus* oocyte system is that proteins may be expressed in variable amounts by injection of corresponding *in vitro* transcribed mRNAs [12]. The DNA reporter is introduced by intranuclear injection of circular single-stranded (ss) DNA, which in our case yielded approximately 600 million gene copies of the MMTV long terminal repeat and all copies are active in terms of specific protein-DNA binding and chromatin remodeling [12]. Importantly, intranuclear injection of ssDNA in *Xenopus* oocytes leads to second-strand DNA synthesis coupled to assembly of a tightly organized chromatin structure [12]. Because of the high copy number of the injected DNA, specific transcription factor-DNA interactions can be quantified with high precision by dimethylsulfate (DMS) *in vivo* footprinting [11], [13]. It is straightforward to isolate the cell nucleus of the oocyte by manual dissection and hence to analyze its protein content.

Here we show that AR with a pathological polyQ tract of 64 residues (ARQ64) has increased capacity for specific DNA binding. Interestingly, this increase did not correlate with an increase in transcription induction at the MMTV promoter. Hence the transcriptional activity of ARQ64 was significantly reduced in comparison to the wild type ARQ25 as related to its DNA binding activity. The possible mechanism for this effect is discussed.

## 2 Materials and methods

### 2.1 Reagents, plasmids and constructs

AR ligands used were R1881 (PerkinElmer Inc., Waltham, MA), as 1×10^−3^ M in EtOH and MDV3100 (enzalutamide) as 1×10^−2^ M in DMSO (from Selleck Chemicals Co. Ltd., Houston, TX). The reporter pMMTV:M13 contains the 1.2 kb MMTV LTR fused to the HSV TK gene and its transfer to M13 was described [12], as has the production of mRNAs. The cDNA coding for the different AR variants were based on pβhAR described before [11], that contains the full length human AR, a kind gift from Dr. Jeming Wong [14]. AR variants with polyQ tracts of different length were generated by restriction cloning. A fragment within AR containing the CAG repeat flanked by *XmaI* and *EcoRI* restriction sites was excised from pβhAR and replaced with fragments containing 0, 13, 25, or 64 CAG repeats. The AR-Q64 cDNA was shown to be contaminated with AR variants containing shorter repeat(s) than Q64. The contaminants were removed by re-transformation of the pβAR-Q64 plasmid in E-coli cells with reduced recombination activity (SURE cells™, Stratagene).

### 2.2 Oocyte injections

**Fig. 1:**
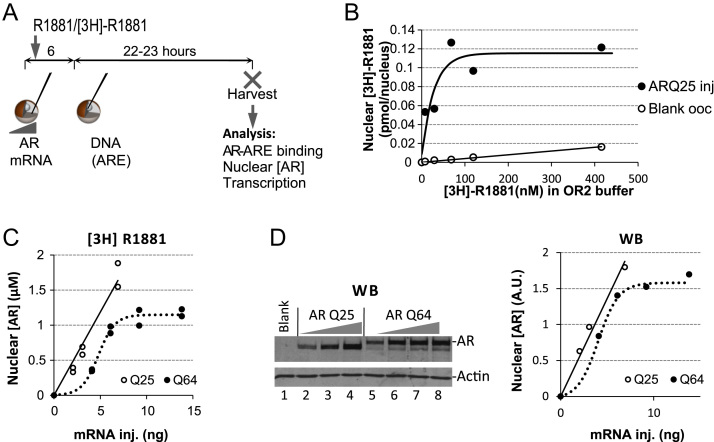
Experimental design and quantification of AR in oocyte nuclei. (A) *Xenopus* oocytes were injected with mRNA into the cytoplasm and with ssDNA into the nucleus and harvested for analysis at indicated time (h). (B) Increasing concentrations of [^3^H]-R1881 were incubated with oocytes either injected or not injected with mRNA coding for ARQ25 and next day taken for analysis of [^3^H]-R1881 in manually isolated nuclei. (C) Oocytes were injected with the indicated amounts of mRNA coding for ARQ25 or ARQ64 and were then analyzed either by quantification of nuclear [^3^H]-R1881 or (D) by Western blot (WB), A.U. indicates arbitrary units.

The animal experiments were conducted according to a defined protocol approved by a local ethical committee (Stockholm N. ethical committee for animal experiments, permit no. N21/12 and N6/15). DNA and mRNA injections into *Xenopus laevis* oocytes have been described previously [15] the protocol is outlined in Fig. 1A. A synthetic androgenic hormone agonist, R1881, was added at 100 nM final concentration, was added immediately after mRNA injection. An aliquot of eight mRNA injected oocytes was incubated in [^3^H]-R1881 for quantification of intranuclear AR (see below). Oocytes were incubated at 21 °C after mRNA injections and until they were harvested ∼28 h later [11].

### 2.3 Quantification of intranuclear AR by specific [^3^H]-R1881 binding

Eight oocytes from each pool of mRNA injected oocytes were placed in a separate 96-well plate containing OR2 buffer [15] and 100 nM concentration of [^3^H]-R1881 (PerkinElmer, 81.2 Ci/mmol). Since *Xenopus* oocytes contain endogenous androgens [16] the concentration of radioactive hormone was titrated to obtain near complete exchange of any endogenous ligand with the radioactively labeled probe this titration showed that [^3^H]-R1881 concentration above 50 nM was enough to fully saturate the expressed AR with radioactive ligand (see results, Fig. 1B). 28 h after mRNA injections, the intranuclear AR protein was analyzed as the radioactivity detectable in isolated oocyte nuclei. A [^3^H]-toluene standard (PerkinElmer) was used to estimate the efficiency for tritium (see results, Fig. 1C). The AR concentration was calculated assuming that one oocyte nucleus has a volume of 40 nl [17]. Routinely, three nuclei were dissected in duplicate for each data point. Nuclear [AR] was defined as the average of the two samples after subtraction of nonspecific radioactivity (see results).

### 2.4 Quantification of the MMTV transcription by S1-nuclease protection and specific DNA binding by DMS in vivo footprinting

Quantification of the MMTV transcription by S1-nuclease protection and specific DNA binding by DMS in vivo footprinting was done as described previously [18]. Nonlinear model fit of data points were generated using the software CurveExpert Pro V.2.2.0. The amount of intranuclear plasmid DNA was recovered as described for DMS *in vivo* footprinting and quantified by primer extension in parallel with a DNA standard [13].

## 3 Results and discussion

### 3.1 ARQ25 and the ARQ64 expression and quantification in Xenopus oocytes

We previously used Western blot (WB) to monitor the amount of AR in *Xenopus* oocytes [11]; an alternative strategy to quantify steroid receptors is to monitor the hormone-receptor complex using a tritiated hormone-ligand as a probe [19]. The latter strategy provides information about the absolute amount of hormone-receptor complex since there is one hormone-binding site in each receptor molecule. *Xenopus* oocytes contain endogenous androgens so it was important to test whether a tritiated hormone ligand could compete efficiently for binding to the receptor in presence of the unlabeled endogenous hormone. Oocytes previously injected with ARQ25 mRNA and incubated with increasing concentration of the tritium labeled androgen agonist R1881 [20] were taken for nuclear dissection. Oocytes not injected with AR mRNA served as negative controls, indicated as blank oocytes in Fig. 1B. Radioactivity analysis of nuclear extracts showed that a [^3^H]-R1881concentration above ∼50 nM efficiently competed for any endogenous ligand since the AR dependent nuclear radioactivity reached a plateau at this level and no further increase occurred even at 400 nM [^3^H]-R1881 (Fig. 1B). The nonspecific radioactivity recovered in blank oocyte nuclei showed a linear relationship with increasing [^3^H]-R1881concentration in the medium. The addition of non-radioactive R1881 together with the tritiated ligand competed efficiently as expected and reduced the nuclear radioactivity accordingly (data not shown).

*Xenopus* oocytes are filled with yolk protein and lipophilic substances. Steroids such as [^3^H]-R1881 are also rather lipophilic. We addressed this potential source of error in quantification of AR based on radioactive ligand by measuring the amount of [^3^H]-R1881 ligand in extracts from whole oocytes and from manually isolated nuclei that were either not injected or injected with ARQ25 mRNA (Supplement 1A). This showed a 0.52 µM concentration of nuclear AR in the oocytes injected with AR mRNA and a signal to noise ratio of 5.1 corresponding to 0.02 pmol of specifically bound hormone ligand per nucleus based on a nuclear volume of 40 nl [17]. The total amount of hormone in the intact oocyte was 1.2–1.5 pmol/cell and thus a 45-fold higher amount than in AR containing nuclei or 260-fold higher than nuclei in oocyte not injected with AR mRNA (Supplement 1A). The level of radioactivity in the cytosol was about the same whether AR was expressed or not and higher than in the media. This illustrates the differential distribution of the lipophilic steroid R1881 in water solution compared to the lipid-containing oocyte cytosol. This was in sharp contrast to the nucleus, where the background level of radioactive ligand was low enough to allow a reproducible quantification of the AR-dependent amounts.

mRNAs coding for the polyQ variants ARQ25 and ARQ64 were injected into the cytoplasm of two different pools of *Xenopus* oocytes and resulting protein expression was analyzed in manually isolated oocyte nuclei either by monitoring [^3^H]-R1881 radioactivity (Fig. 1C) or by WB analysis of nuclear extracts (Fig. 1D). Both methods show that the stepwise increase of injected of ARQ25 mRNA rendered a linear increase of expressed ARQ25 protein (Fig. 1C and D). However, the ARQ64 expression generated by mRNA amounts above 6 ng per oocyte reached a plateau indicating that saturation in protein expression was achieved (see Fig. 1C and D). This comparison between the quantification of nuclear AR based on retained [^3^H]-R1881 and WB showed the two methods to correlate well. However, we found that WB tends to produce more variable results and furthermore the WB method did not provide information about absolute amounts of receptor. Both methods have been used in this work, but we favor the [^3^H]-R1881-based approach for intranuclear AR quantification.

**Fig. 2:**
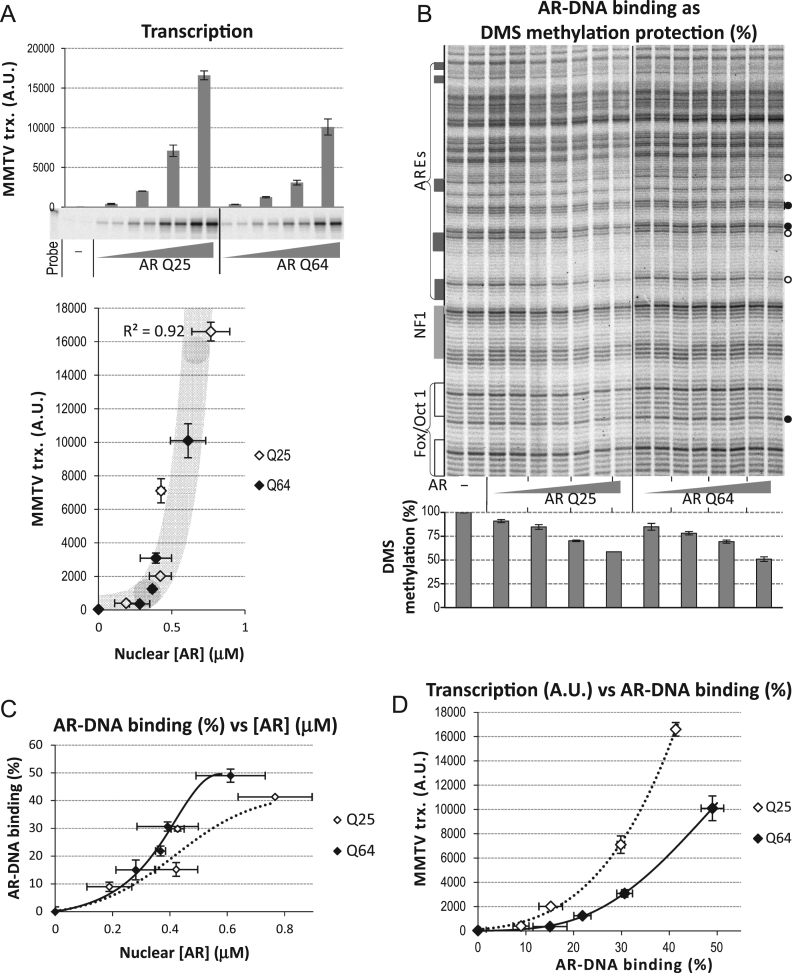
Comparison of ARQ25 and ARQ64. (A) Quantification of MMTV transcription by S1 nuclease protection analysis of oocytes injected with either 2.1; 3.1; 4.6 or 6.9 ng ARQ25 mRNA 2.7; 4.1; 6.1 or 9.2 ng of ARQ64 mRNA and then 3 ng ssDNA pMMTV:M13 and exposed to 100 nM R1881. A.U. indicates arbitrary units. The diagram below shows MMTV transcription as a function of nuclear [AR](µM) based on [^3^H]-R1881 analysis. Gray shadow indicates the curve fitting of all data by software Curve Expert Pro v.2.2.0. (B) Autoradiogram of primer extension from DMS *in vivo* footprinting of aliquots of the same oocytes as in Fig. 2A. Specific DNA sites for AR (ARE) are indicated on the left side together with binding sites for other proteins (not expressed here), radioactive bands protected in presence of AR, *i.e.* DMS methylation protected, are marked to the right with empty circles and reference bands for loading control as filled circles. Quantification of the average value of the protected bands, two lanes per oocyte pool, is shown as columns below with the average deviation of double samples as error bars. The last lane was lost in ARQ25. (C) AR-DNA binding, based on DMS methylation protection, plotted as a function of intranuclear [AR], based on quantification of nuclear [^3^H]-R1881. The curves are calculated based on the Curve Expert Pro v 2.2.0 software. (D) Transcription of MMTV RNA analyzed by S1 nuclease was plotted as a function of AR-DNA binding activity from DMS *in vivo* footprinting.

One problem that we encountered was that the ARQ64 expressed protein migrates on SDS PAGE as a double band. The faster moving band constituted 10–25% of the total signal (cf. Fig. 1D, lanes 5–8) and was not present in other AR variants with smaller polyQ length which always appeared as a single band (cf Fig. 1D, lanes 2–4). The faster migrating band of ARQ64 showed an electrophoretic mobility similar to ARQ25 (Fig. 1D). Restriction enzyme analysis of the cDNA of ARQ64 as well as WB using different AR antibodies, and in addition one antibody directed to long polyQ repeats [21], indicated that the ARQ64 plasmid used for *in vitro* mRNA transcription was contaminated by an AR variants containing shorter polyQ repeats of a similar size as ARQ25 (data not shown and Supplement 1B). Since DNA-repetitive sequences are prone to be deleted by recombination during amplification in E-coli, we re-amplified the ARQ64 cDNA from a single colony using E-coli with reduced recombination capacity (Sure Cells™, Stratagene). RNA produced from this plasmid that was injected into oocytes gave rise to a single band of ARQ64 by WB (Supplement 1B, compare lanes 2 and 4). This purified ARQ64 template was used in most of the experiments comparing ARQ64 to ARQ25 (c.f. Fig. 2).

### 3.2 Functional comparison of ARQ25 and ARQ64

Groups of oocytes were injected with increasing amounts of mRNA coding for ARQ25 or ARQ64 as indicated (Fig. 1A) followed by 3 ng of ssDNA of pMMTV:M13 harboring the MMTV enhancer and promoter. Aliquots of oocytes from each group were collected after mRNA injection and incubated with ∼100 nM [^3^H]-R1881 and after 28 h the oocytes were harvested and analyzed for nuclear AR concentration, AR-driven MMTV transcription (Fig. 2A), and sequence specific AR-DNA binding (Fig. 2B) by DMS *in vivo* footprinting.

A comparison of the two AR variants did not show any difference in the capacity to induce MMTV transcription when related to the nuclear AR concentration (see Fig. 2A, lower diagram). However, the sequence-specific DNA binding, defined as DMS methylation protection (Fig. 2B, see protected bands marked with open circles) plotted as a function of nuclear AR concentration (Fig. 2C) demonstrated a stronger DNA binding by ARQ64. Importantly, a diagram showing AR-driven transcription as a function of AR-DNA binding activity revealed a 2.5-fold increased transcriptional response for ARQ25 as compared to ARQ64 (Fig. 2D). This result was reproduced twice (c.f. Supplement 2). In all three experiments a more robust transcriptional response was seen by ARQ25 as compared to ARQ64 in relation to its specific DNA binding activity. These results argue for a functional dissociation between the DNA binding event and the transcriptional induction seen with the ARQ64 variant.

### 3.3 No difference between ARQ25 and ARQ64 in androgen agonist- or antagonist-dependent nuclear translocation

**Fig. 3:**
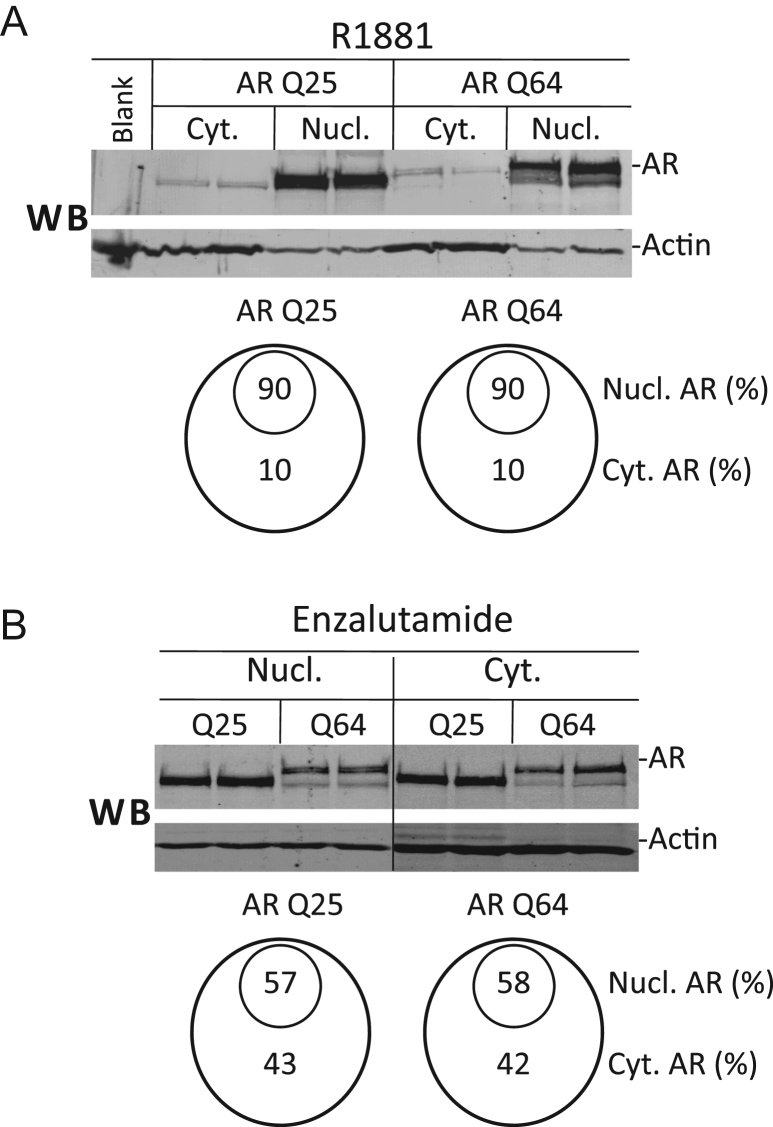
Comparison of nuclear and cytosolic distribution of ARQ25 and ARQ64 (A) in presence of androgen agonist R1881 or (B) androgen antagonist enzalutamide. Oocytes were injected with 3.5 ng ARQ25 mRNA or 6.9 ng of ARQ64 mRNA followed by 3 ng ssDNA pMMTV:M13 as in Fig. 1A. 28 h later oocytes were harvested and processed for SDS PAGE and WB (see Section 2). 0.75 of oocyte equivalent of cytosol or nuclear extract was applied on each lane. The smaller ARQ64 sub-band of MW ∼120 kDa was also present in this experiment since the re-amplification of the pβARQ64 described above was done later. The ratio of the main band and the smaller band of ARQ64 remained constant when comparing cytosolic and nuclear ARQ64 (data not shown).

The AR is transported into the cell nucleus in a hormone-dependent fashion. The kinetics of the hormone-dependent nuclear translocation could not be addressed since *Xenopus* oocytes contain endogenous androgens. Instead we analyzed the hormone-dependent nuclear localization with the ARQ25 and ARQ64 variants at equilibrium, *i.e.* 6 h after addition of the agonist, R1881, or an antagonist, enzalutamide; the latter is an antiandrogen that has been shown to reduce nuclear translocation of AR in *Xenopus* oocytes [11]. Oocytes injected with mRNA coding for either ARQ25 or ARQ64 were incubated either with 5 nM R1881 or 1 µM enzalutamide and the nuclear and cytosolic extracts were analyzed by WB. This showed a strong tendency for the AR to be localized in the nucleus in the presence of the agonist R1881, where 90% of total AR was nuclear for both AR variants (Fig. 3A), as well as a distinct reduction of the nuclear uptake in the presence of enzalutamide, where about 58% was nuclear (Fig. 3B) for both AR variants. Hence the androgen antagonist caused about a four-fold increase in the percentage of cytosolic AR for both AR variants.

We conclude that there is no difference in the nuclear and cytoplasmic distribution of the ARQ25 and the ARQ64. The experiment also indicates that the ligand binding specificity for the ligands tested here is the same for both AR variants.

### 3.4 AR with shorter polyQ repeats, ARQ0 and ARQ13, showed no difference in transcriptional activity compared to ARQ25

Our finding of a reduced capacity by ARQ64 to drive MMTV transcription as related to its specific DNA binding activity (c.f. Fig. 2D) encouraged us also to address the biological activity of AR with shorter polyQ repeats; hence we developed ARQ0 and ARQ13 constructs. The *in vitro* transcribed mRNA was injected into oocytes and 28 h later nuclei and cytosol was separated by manual dissection and the relative amounts of AR in nuclear and cytosol extracts estimated by WB. This indicated an average nuclear AR localization of 98, 97 and 96% for ARQ0; Q13 and Q25, respectively. Thus there was no difference in nuclear uptake between AR25Q and AR variants with shorter polyglutamine tracts (data not shown).

Oocytes injected with the three AR variants Q0, Q13 and Q25 followed by the ssDNA injection, 3 ng MMTV: M13, three independent experiments did not generate any consistent difference in expression (Supplement 3B), DNA binding or transcription (Appendix A, Appendix A) when relating these activities to the relative intranuclear AR levels, here monitored by WB. We conclude that there was no major difference in AR function for the AR variants containing non-pathological polyQ repeats, suggesting that the effects observed for ARQ64 are specific for the polyQ-expanded AR. However, this finding does not exclude functional differences of AR with shorter repeats in another promoter- or cellular context. As mentioned above, both increased [8] and decreased [5] transcriptional activity have been reported with increasing length of the polyQ repeat.

### 3.5 Final remarks

We observed a difference in sequence-specific DNA binding by ARQ64. Unexpectedly, this AR variant showed increased capacity to bind specific DNA sequence in a chromatinized template *in vivo.* This difference was distinct when comparing DNA binding as a function of nuclear AR concentration (Fig. 2C and Supplement 2C), but it was even more robust when relating the DNA binding to its capacity to induce transcription (Fig. 2D and Supplement 2D). In the latter case the ARQ25 had a much stronger capacity to induce transcription than the ARQ64 variant. Importantly, this difference was not apparent when relating MMTV transcription to the nuclear AR concentration (Fig. 2A, lower diagram and Supplement 2, lower diagram). This is unexpected since all steroid receptors show hormone-dependent specific DNA binding activity as an important step in the chain of events involved in the hormone-driven gene induction. It suggests that ARQ64 has a reduced capacity to convert the DNA binding event into a transcriptional response.

Interestingly, inhibiting SUMOylation of AR with an expanded polyQ repeat counteracted the significant loss of transactivation caused by polyQ expansion and ameliorated the mutant AR-mediated disease in a mouse model [10]. A deubiquitinating enzyme Usp12 was recently shown to act as a coactivator for AR in prostate cancer cells [22]. If such modifications occur in oocytes and play a role in the process involved in converting a DNA binding event into a transcriptional response then the elongated polyQ of the ARQ64 variant might reduce or alter this process. We speculate that posttranslational modifications are changed in the context of an extended polyQ and that this contributes to the reduced transcriptional response with ARQ64.

We are the first to report increased specific DNA binding capacity of the ARQ64 *in vivo*. This important step in the mechanism of action of androgens is confined to the chromatin target and the transactivating factor, *i.e.* AR. It remains to be determined whether this result also applies to other AR driven enhancers and in other cellular context.

## Conflict of interest

The authors have declared no conflicts of interest [23].

## Footnotes

Appendix A Supplementary data associated with this article can be found in the online version at doi:10.1016/j.bbrep.2015.07.014.
