# Supplementary material for: The polyglutamine-expanded androgen receptor has increased DNA binding and reduced transcriptional activity
Source: Biochem Biophys Rep. 2015 Jul 26;3:134–9. doi: 10.1016/j.bbrep.2015.07.014 (PMC5668691; doi:10.1016/j.bbrep.2015.07.014)
Supplement: Supplementary file 1 — Supplementary material [file mmc1.pptx]

## Slide 1
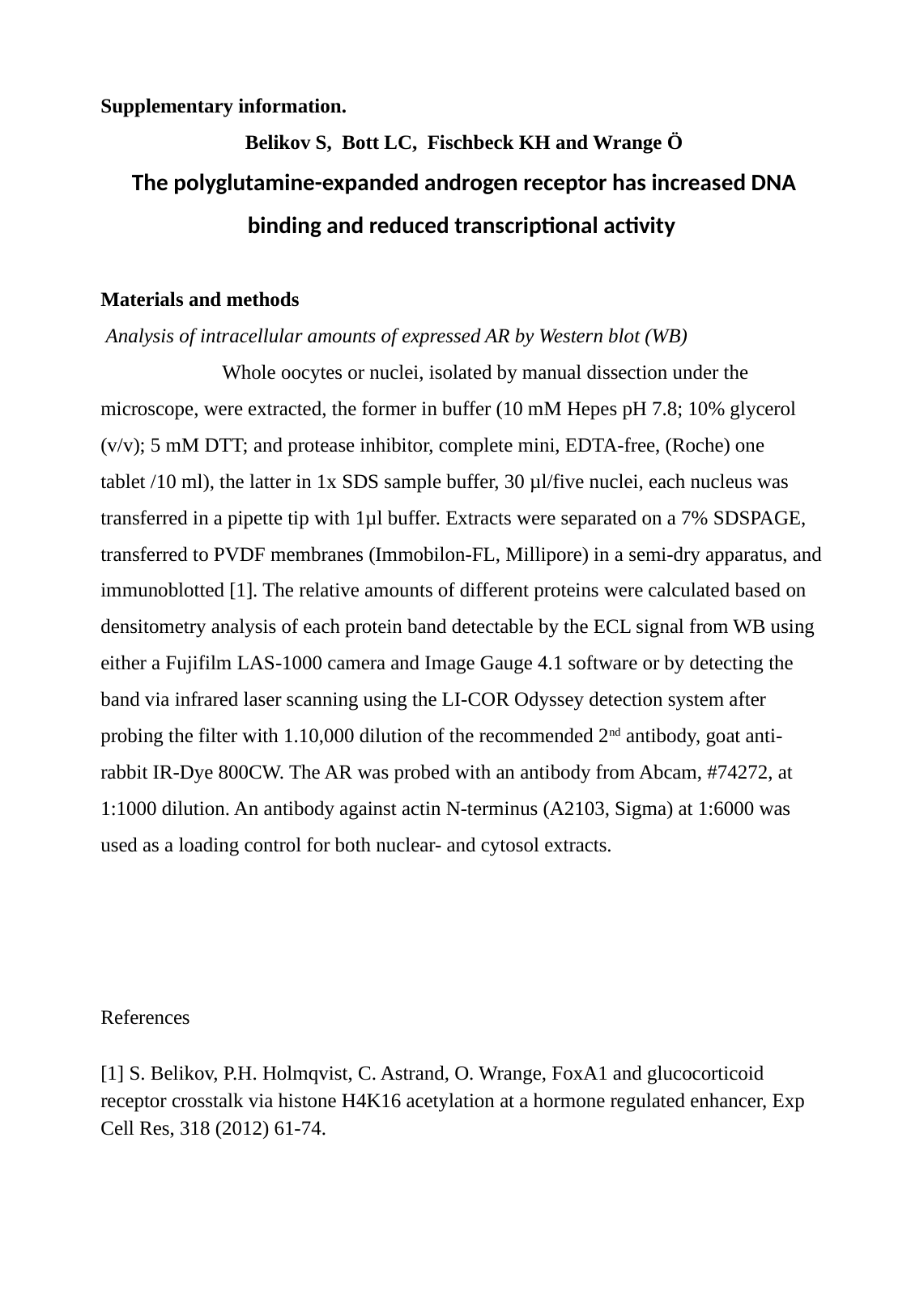

Supplementary information.
Belikov S, Bott LC, Fischbeck KH and Wrange Ö
The polyglutamine-expanded androgen receptor has increased DNA binding and reduced transcriptional activity
Materials and methods
 Analysis of intracellular amounts of expressed AR by Western blot (WB)
	Whole oocytes or nuclei, isolated by manual dissection under the microscope, were extracted, the former in buffer (10 mM Hepes pH 7.8; 10% glycerol (v/v); 5 mM DTT; and protease inhibitor, complete mini, EDTA-free, (Roche) one tablet /10 ml), the latter in 1x SDS sample buffer, 30 µl/five nuclei, each nucleus was transferred in a pipette tip with 1µl buffer. Extracts were separated on a 7% SDSPAGE, transferred to PVDF membranes (Immobilon-FL, Millipore) in a semi-dry apparatus, and immunoblotted [1]. The relative amounts of different proteins were calculated based on densitometry analysis of each protein band detectable by the ECL signal from WB using either a Fujifilm LAS-1000 camera and Image Gauge 4.1 software or by detecting the band via infrared laser scanning using the LI-COR Odyssey detection system after probing the filter with 1.10,000 dilution of the recommended 2nd antibody, goat anti-rabbit IR-Dye 800CW. The AR was probed with an antibody from Abcam, #74272, at 1:1000 dilution. An antibody against actin N-terminus (A2103, Sigma) at 1:6000 was used as a loading control for both nuclear- and cytosol extracts.
References
[1] S. Belikov, P.H. Holmqvist, C. Astrand, O. Wrange, FoxA1 and glucocorticoid receptor crosstalk via histone H4K16 acetylation at a hormone regulated enhancer, Exp Cell Res, 318 (2012) 61-74.

## Slide 2
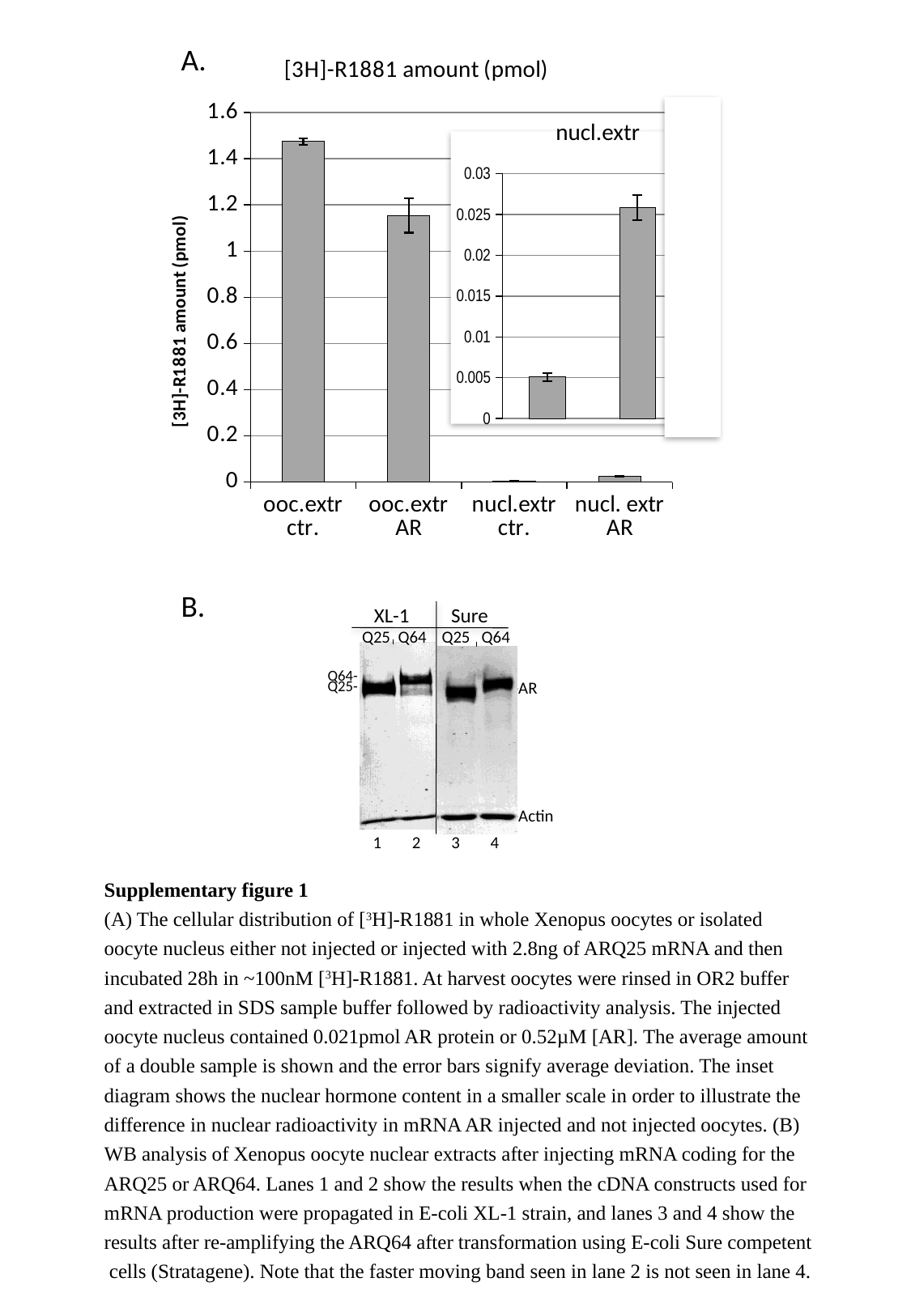

A.
B.
### Chart: [3H]-R1881 amount (pmol)
| Category | |
|---|---|
| ooc.extr ctr. | 1.475 |
| ooc.extr AR | 1.155 |
| nucl.extr ctr. | 0.00508809637120761 |
| nucl. extr AR | 0.0258419095776324 |
nucl.extr
### Chart
| Category | |
|---|---|
| nucl.extr ctr. | 0.00508809637120761 |
| nucl. extr AR | 0.0258419095776324 |XL-1 Sure
Q25 Q64 Q25 Q64
Q64-
Q25-
-AR
-Actin
1 2 3 4
Supplementary figure 1
(A) The cellular distribution of [3H]-R1881 in whole Xenopus oocytes or isolated oocyte nucleus either not injected or injected with 2.8ng of ARQ25 mRNA and then incubated 28h in ~100nM [3H]-R1881. At harvest oocytes were rinsed in OR2 buffer and extracted in SDS sample buffer followed by radioactivity analysis. The injected oocyte nucleus contained 0.021pmol AR protein or 0.52µM [AR]. The average amount of a double sample is shown and the error bars signify average deviation. The inset diagram shows the nuclear hormone content in a smaller scale in order to illustrate the difference in nuclear radioactivity in mRNA AR injected and not injected oocytes. (B) WB analysis of Xenopus oocyte nuclear extracts after injecting mRNA coding for the ARQ25 or ARQ64. Lanes 1 and 2 show the results when the cDNA constructs used for mRNA production were propagated in E-coli XL-1 strain, and lanes 3 and 4 show the results after re-amplifying the ARQ64 after transformation using E-coli Sure competent cells (Stratagene). Note that the faster moving band seen in lane 2 is not seen in lane 4.

## Slide 3
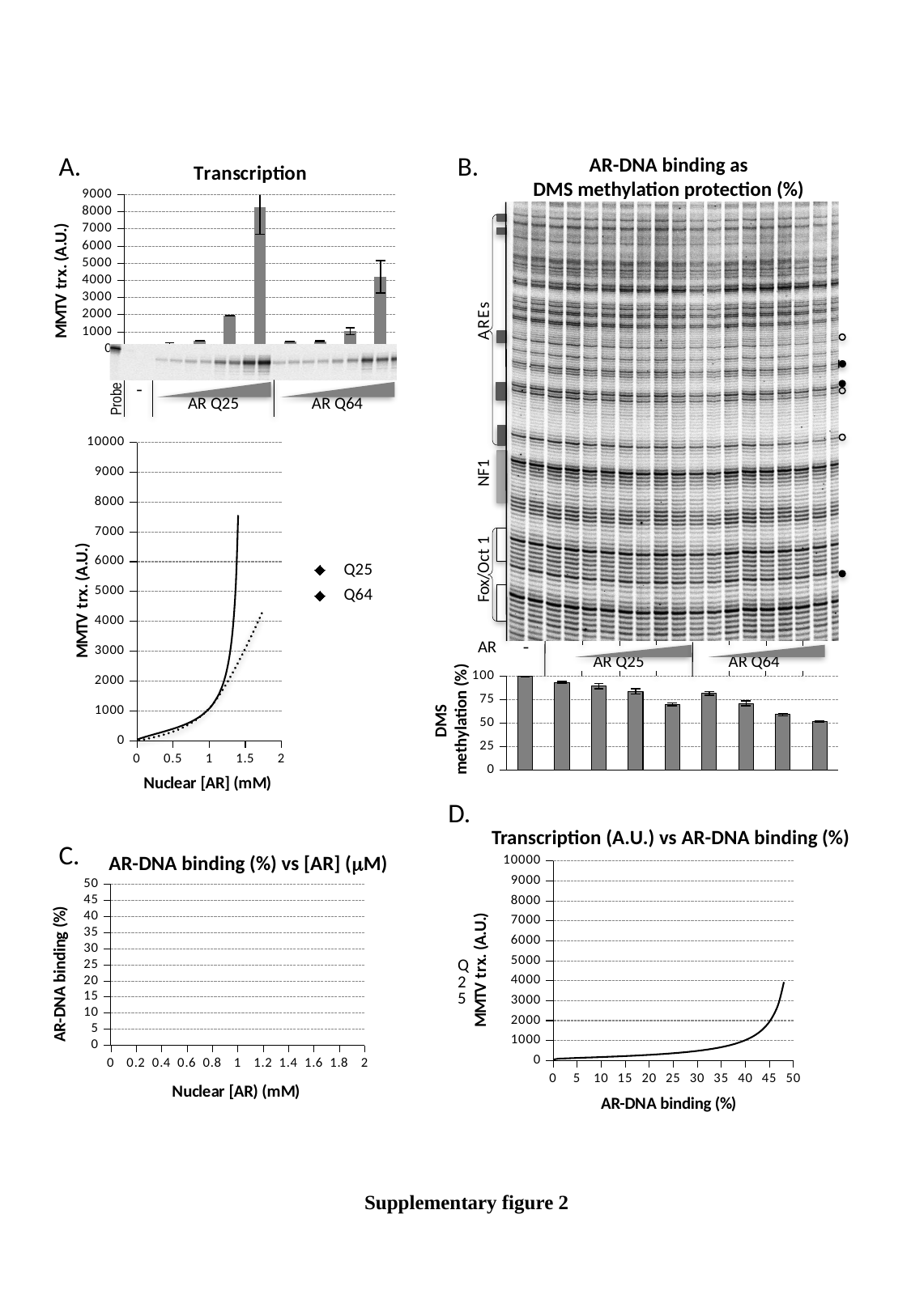

A. B.
 D.
C.
AR-DNA binding as
DMS methylation protection (%)
### Chart
| Category | MMTV trx |
|---|---|
### Chart
| Category | | | |
|---|---|---|---|
Fox/Oct 1 NF1 AREs
 -
 AR Q25 AR Q64
Probe
AR -
 AR Q25 AR Q64
### Chart
| Category | ARE, % |
|---|---|DMS
methylation (%)
Transcription (A.U.) vs AR-DNA binding (%)
AR-DNA binding (%) vs [AR] (mM)
### Chart
| Category | | | | |
|---|---|---|---|---|
### Chart
| Category | | | | |
|---|---|---|---|---|Supplementary figure 2

## Slide 4
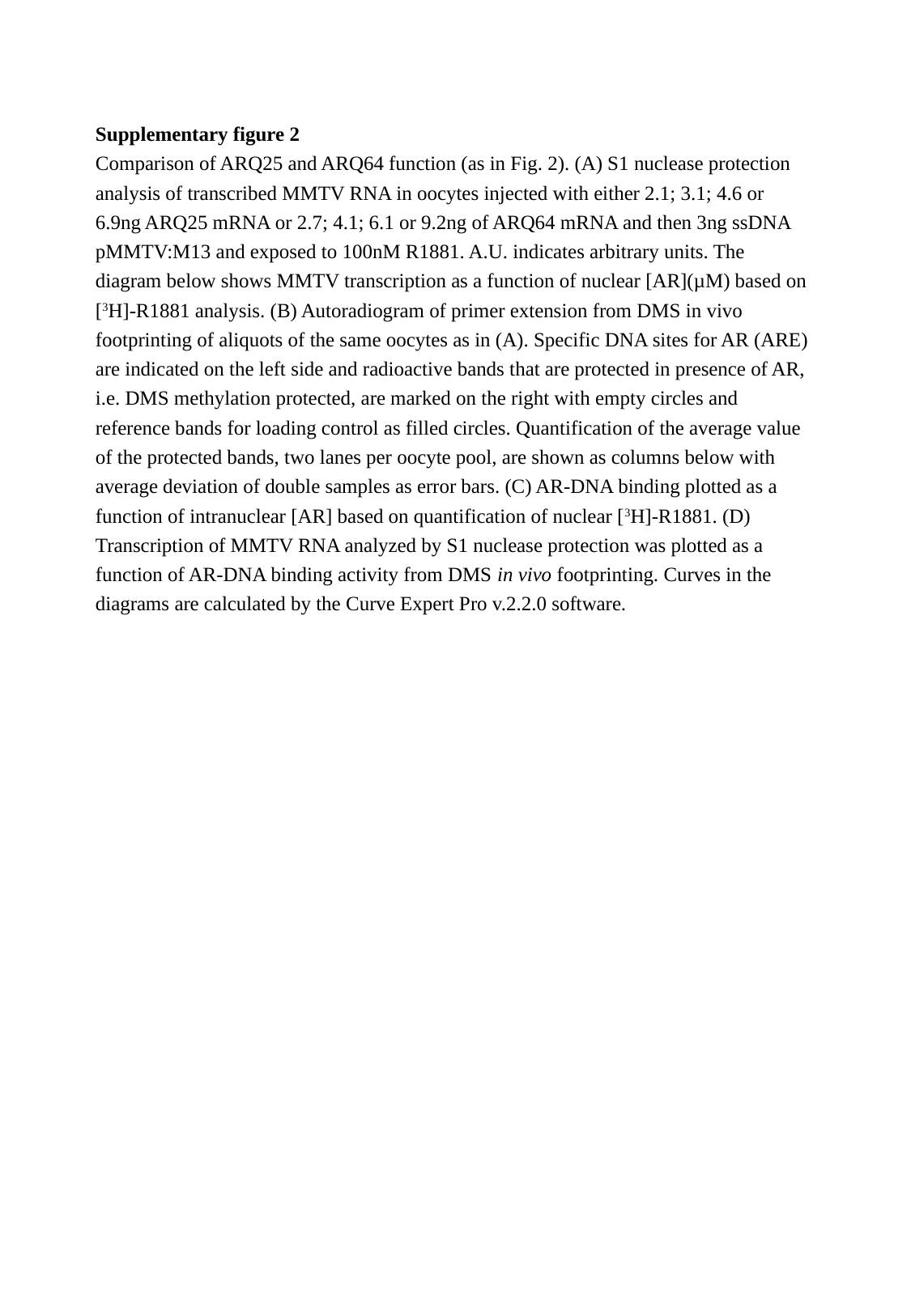

Supplementary figure 2
Comparison of ARQ25 and ARQ64 function (as in Fig. 2). (A) S1 nuclease protection analysis of transcribed MMTV RNA in oocytes injected with either 2.1; 3.1; 4.6 or 6.9ng ARQ25 mRNA or 2.7; 4.1; 6.1 or 9.2ng of ARQ64 mRNA and then 3ng ssDNA pMMTV:M13 and exposed to 100nM R1881. A.U. indicates arbitrary units. The diagram below shows MMTV transcription as a function of nuclear [AR](µM) based on [3H]-R1881 analysis. (B) Autoradiogram of primer extension from DMS in vivo footprinting of aliquots of the same oocytes as in (A). Specific DNA sites for AR (ARE) are indicated on the left side and radioactive bands that are protected in presence of AR, i.e. DMS methylation protected, are marked on the right with empty circles and reference bands for loading control as filled circles. Quantification of the average value of the protected bands, two lanes per oocyte pool, are shown as columns below with average deviation of double samples as error bars. (C) AR-DNA binding plotted as a function of intranuclear [AR] based on quantification of nuclear [3H]-R1881. (D) Transcription of MMTV RNA analyzed by S1 nuclease protection was plotted as a function of AR-DNA binding activity from DMS in vivo footprinting. Curves in the diagrams are calculated by the Curve Expert Pro v.2.2.0 software.

## Slide 5
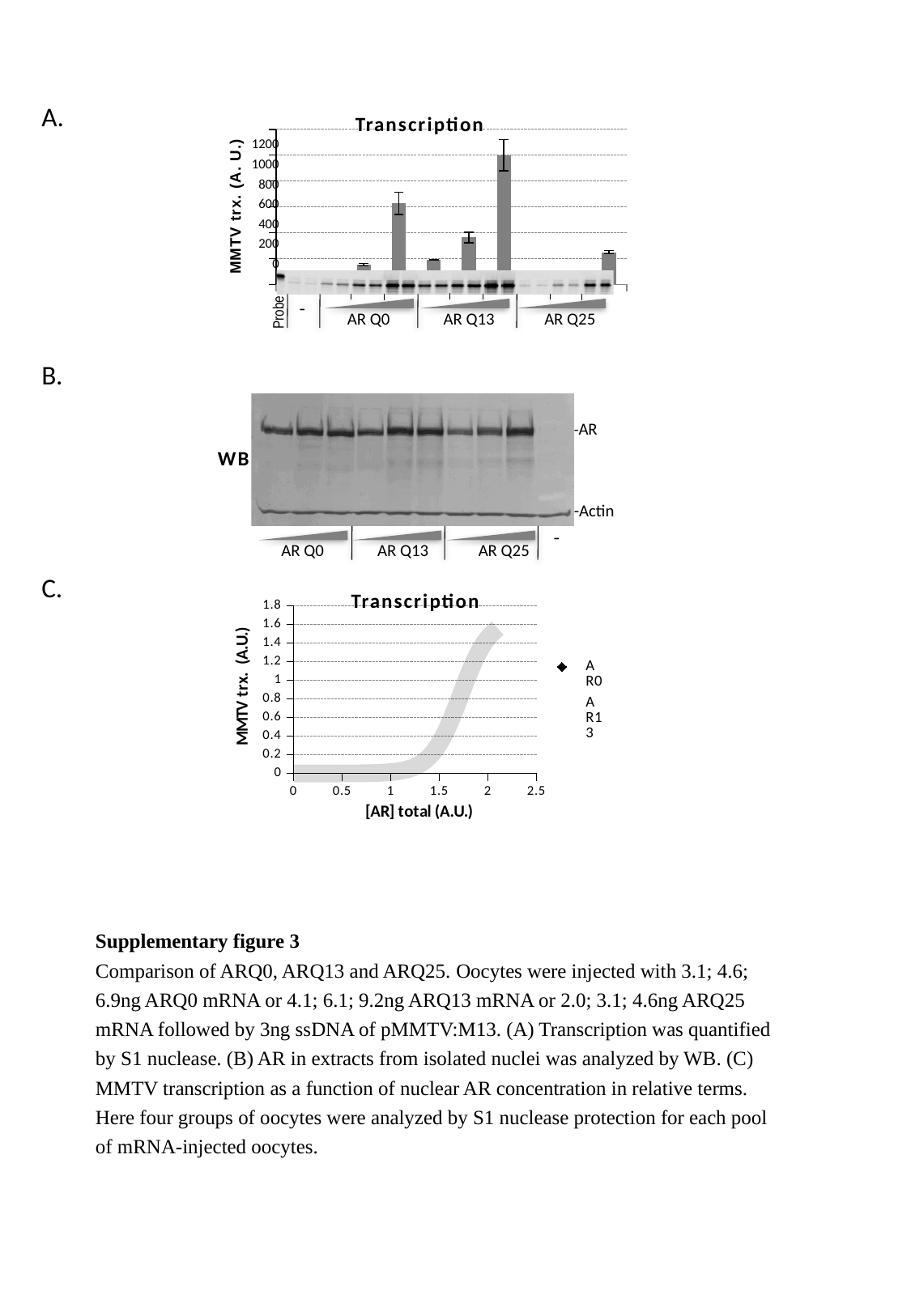

A.
B.
C.
Transcription
### Chart
| Category | MMTV |
|---|---|1200
1000
800
600
400
200
0
MMTV trx. (A. U.)
-
Probe
 AR Q0 AR Q13 AR Q25
-
 AR Q0 AR Q13 AR Q25
-AR
-Actin
WB
Transcription
### Chart
| Category | | | | |
|---|---|---|---|---|Supplementary figure 3
Comparison of ARQ0, ARQ13 and ARQ25. Oocytes were injected with 3.1; 4.6; 6.9ng ARQ0 mRNA or 4.1; 6.1; 9.2ng ARQ13 mRNA or 2.0; 3.1; 4.6ng ARQ25 mRNA followed by 3ng ssDNA of pMMTV:M13. (A) Transcription was quantified by S1 nuclease. (B) AR in extracts from isolated nuclei was analyzed by WB. (C) MMTV transcription as a function of nuclear AR concentration in relative terms. Here four groups of oocytes were analyzed by S1 nuclease protection for each pool of mRNA-injected oocytes.
